# Peri‐left bundle branch pacing after atrioventricular node ablation and failed his bundle pacing in atrial fibrillation

**DOI:** 10.1002/joa3.12299

**Published:** 2020-01-08

**Authors:** Alexander Edo Tondas, Raymond Pranata, Han Hongwei

**Affiliations:** ^1^ Department of Cardiology and Vascular Medicine Mohammad Hoesin General Hospital Palembang Sumatera Selatan Indonesia; ^2^ Biomedicine Doctoral Program Faculty of Medicine Universitas Sriwijaya Palembang Indonesia; ^3^ Faculty of Medicine Universitas Pelita Harapan Tangerang Banten Indonesia; ^4^ Department of Cardiovascular Medicine Wuhan Asia Heart Hospital Wuhan China

**Keywords:** atrial fibrillation, catheter ablation, His bundle pacing, peri‐left bundle branch pacing, rate control

## Abstract

We described a case where peri‐left bundle branch pacing (PLBP) may become an alternative approach in difficult His bundle pacing (HBP) following atrioventricular nodal ablation in a patient with atrial fibrillation. After atrioventricular nodal ablation, the HBP lead was removed to another LBB position distal to the first PLBP lead, due to acute threshold increase. At 3 month follow‐up, PLBP exhibited acceptable pacing parameters without any adverse event.

## Graphical abstract

We described a case where peri‐left bundle branch pacing (PLBP) may become an alternative approach in difficult His bundle pacing (HBP) following atrioventricular nodal ablation in a patient with atrial fibrillation. After atrioventricular nodal ablation, the HBP lead was removed to another LBB position distal to the first PLBP lead, due to acute threshold increase. At 3 months follow‐up, PLBP exhibited acceptable pacing parameters without any adverse event.

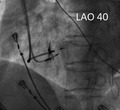

## 1 INTRODUCTION

His bundle pacing (HBP) had emerged as a novel attractive method of physiological pacing associated with a better clinical outcome compared to conventional right ventricular pacing. However, some challenges such as higher pacing threshold and difficulty mapping the His bundle area still remain. In this case report we described peri‐left bundle branch pacing (PLBP) as a bail out technique for unsuccessful HBP after AV nodal ablation.

## 2 CASE REPORT

A 67‐year‐old female, was admitted with symptomatic uncontrolled AF rapid ventricular response and preserved left ventricular ejection fraction (LVEF) of 50%. Echocardiographic measurements showed enlarged left and right atrium, with diameters of 45 mm and 49 mm, respectively. Left ventricle (LV) and right ventricle (RV) dimensions were in normal range.

**Figure 1:**
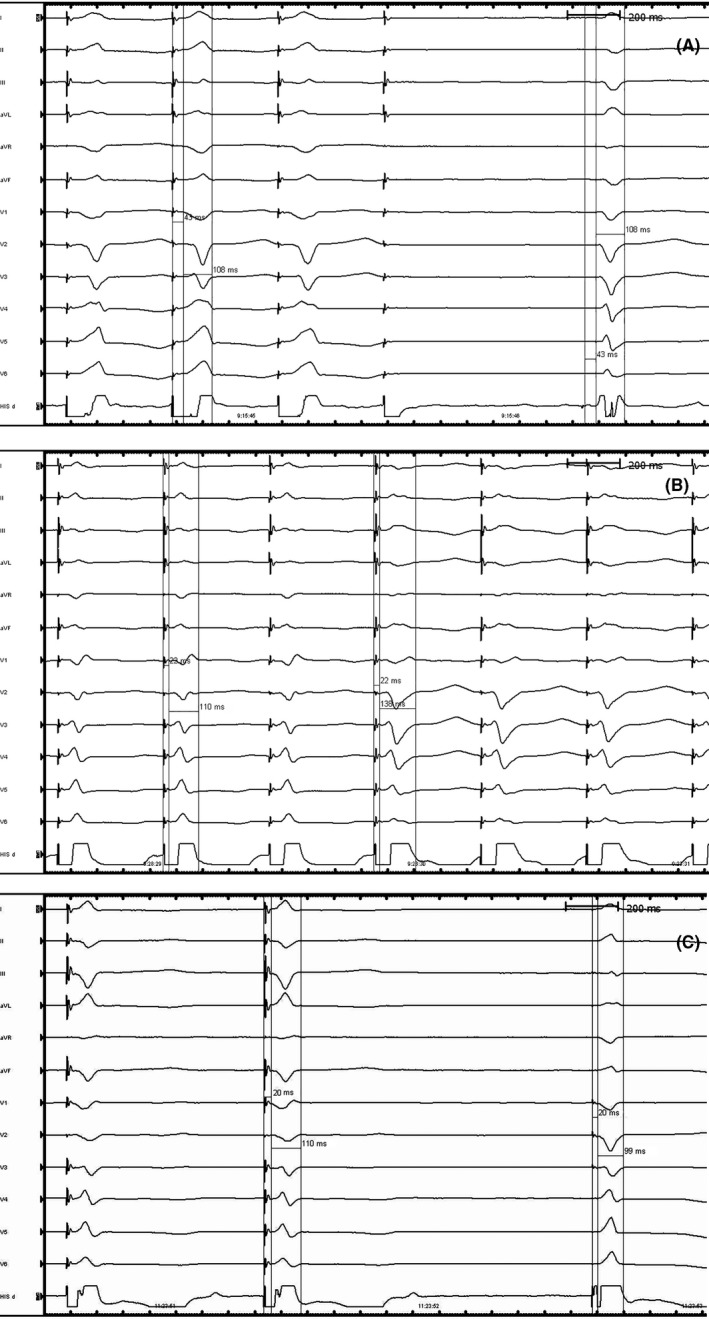
Cardiac electrogram recording. First cardiac electrogram demonstrated anNonselective His bundle pacing (before ablation) HV = 43 msec and QRS duration = 108 ms (A). Second cardiac electrogram showed transition from nonselective proximal PLBP during high output unipolar pacing to selective at 1.0 V/0.4 ms. PV interval = 22 ms (B). Third cardiac electrogram demonstrated a nonselective distal PLBP pacing with PV = SV interval = 20 ms and QRS duration 110 ms (C)

**Figure 2:**
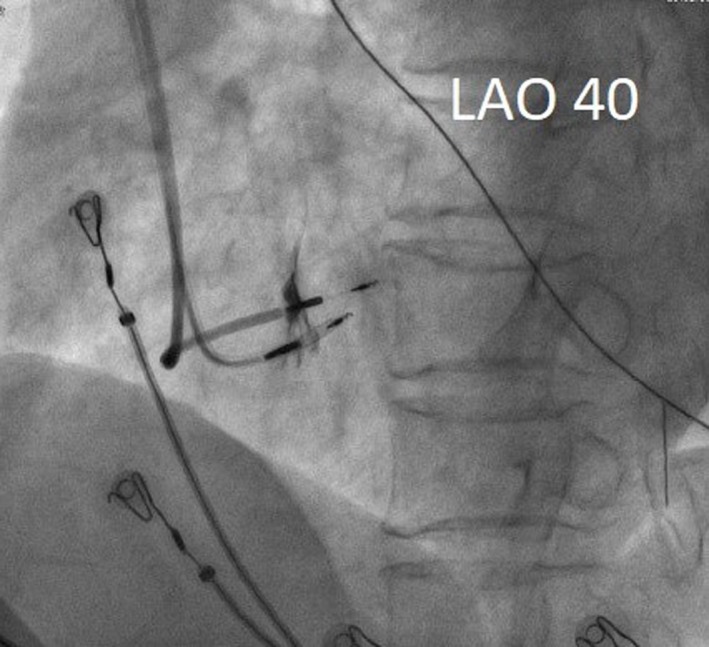
After AV nodal ablation, HBP pacing lead was removed to peri‐left bundle branch position distal to the first PLBP lead and screwed‐in around 12 mm intraseptally (LAO 40° view)

Nonselective HBP was achieved using a Select Secure 3830 His lead with a threshold of 1.4 V/0.4 ms (Figure 1A). The detailed step‐by‐step technique for HBP has been previously described elsewhere.^1^ Another Select Secure 3830 His lead was placed in LBB position. Fluoroscopically, LBB will usually be found at around 1‐1.5 cm distance toward the apex from the previously marked HB EGM, as previously suggested by the literature.^2^ The lead was then fixated in LBB, trans‐ and intraseptally using 15‐20 clockwise torque turns (Figure 2), guided by contrast injections until Purkinje fiber potential can be recorded in the EGM. PLBP showed W‐shaped notch to QR shape (RBBB morphology) on V1 ECG lead during screw‐in, indicating LBB capture. Transition from nonselective to selective unipolar LBB pacing was observed at 1.0 V/0.4 ms, with paced QRS wave durations of 114 msec and 136 msec, respectively. A threshold of 0.5 V/0.4 ms was accepted. PV (Purkinje fiber potential to V wave) interval was 22 msec (Figure 1B).

AV node ablation was then performed, resulting in junctional escape rhythm of 50 beats per minute and QRS duration and HV interval were 108 msec and HV 43 ms respectively. We were only able to achieve complete AV block with the ablation catheter at a distance less than 8 mm from the pacing site, after several failed attempts. During retesting, it turned out that HBP threshold had increased to 6 V/0.4 ms. HBP lead was removed and replaced to another LBB position distal to the first PLBP, yielding a threshold of 1.0 V/0.4 ms. Nonselective distal LBB pacing resulted in narrow QRS duration (110 msec) and similar PV to SV (stimulus to V wave) interval of 20 ms (Figure 1C). Proximal PLBP lead with better threshold was connected to the atrial port and acted as the primary DDD pacing of 75×/min. By connecting the distal PLBP to the ventricular port, a backup pacing configuration was set after a certain AV delay setting (150 ms).

The patient was asymptomatic and discharged without adverse events. Follow‐up echocardiography showed no indication of new‐onset tricuspid regurgitation and the patient's NYHA class had improved from Class III before proceeding to Class II, 3 months after the procedure. There was also a slight increment of LVEF to 54%. Pacemaker interrogation at 1 year after implant showed acceptable threshold of both proximal and distal PLBP at 0.75 V/0.4 msec.

## 3 DISCUSSION

First experimented in humans by Deshmukh in the year 2000 and revived by Vijayaraman et al in 2015, His bundle pacing (HBP) has gained popularity in recent years in pursuit of “physiological pacing”. Nowadays the implantation success rate ranged at >80%–92% after learning curve with the introduction of the 3830 *Select Secure* lead.^1^

Despite the prospective ability to correct bundle branch blocks and maintenance of narrow QRS complex, HBP was confronted with several limitations. In patients with advanced heart block, HBP was successful in 93% of AV nodal block and in infra‐nodal block patients, the success rate is lower at 76%.^1^ Moreover, Vijayaraman reported acute threshold increase of 0.5‐1.5 V after AV nodal ablation in the presence of HBP lead, especially when performed near the tip‐electrode or between the tip and ring. Consequently, an RV backup pacing becomes imperative, especially in those with advanced His‐purkinje disease.^3^

Recently Huang et al had shown the feasibility of pacing the LBB region in a patient with LBB block.^4^ Another case report tried to observe the effect of HBP and PLBP on electrical activation using electrocardiographic imaging in a patient previously with left bundle branch block. Both selective His bundle pacing (SHBP) and PLBP demonstrated synchronous activation of the LV without a line of conduction block with shorter LV and global activation time compared to intrinsic rhythm.^5^ This finding suggested the prospective ability of PLBP to correct bundle branch blocks, similar to HBP.

Through this case, we would like to propose that PLBP may become a solution in difficult HBP pacing. We switched the strategy from HBP and proximal PLBP (backup) to proximal PLBP and distal PLBP (backup). Wide separation of pacing leads was pursued fluoroscopically by directing the proximal PLBP into the proximity of left anterior fascicle (LAF) and the distal PLBP to left posterior fascicle (LPF) anatomical area. The distinction of pacing in different fascicles of the left bundle branch has yet to be elucidated in future studies.

## 4 CONCLUSION

Peri‐left bundle branch pacing can be an alternative to his bundle pacing with similar “physiological pacing” concept. In this case, we successfully attempted sequential PLBP following the failure of HBP after AV nodal ablation for rate control strategy in AF. Although acute pacing parameters were satisfactory with the maintenance of narrow paced QRS complex, future observations should address the long‐term outcome of this method, including the issue of lead extraction.

## CONFLICT OF INTEREST

The authors declare no conflict of interests for this article.

## AUTHOR CONTRIBUTION

HH admitted and treated the patient. AET drafted the manuscript. AET and RP performed extensive research on the topic. HH made critical revisions to the manuscript. All authors contributed to the design of the manuscript. All authors read and approved the final manuscript.
